# Curcumin clarithromycin nano-form a promising agent to fight *Helicobacter* *pylori* infections

**DOI:** 10.1007/s11274-023-03745-7

**Published:** 2023-09-29

**Authors:** Farag M. Mosallam, Mahmoud M. Bendary, Rana Elshimy, Ahmed I. El-Batal

**Affiliations:** 1 Drug Radiation Research Department, Microbiology Lab, Biotechnology Division, National Center for Radiation Research and Technology (NCRRT), Egyptian Atomic Energy Authority, Cairo, Egypt; 2 Microbiology and Immunology Department, Faculty of pharmacy, Port-Said University, Port Fuad, Egypt; 3 Microbiology and immunology, Faculty of pharmacy, AL-Aharm Canadian University (ACU), Giza, Egypt; 4 Egyptian Drug Authority, EDA, Cairo, Egypt

**Keywords:** *H. pylori*, Nano-emulsion, Curcumin, Clarithromycin, In vitro and in vivo antimicrobial

## Abstract

*Helicobacter pylori* (*H. pylori*) is the main cause of gastric diseases. However, the traditional antibiotic treatment of *H. pylori* is limited due to increased antibiotic resistance, low efficacy, and low drug concentration in the stomach. This study developed a Nano-emulsion system with ability to carry Curcumin and Clarithromycin to protect them against stomach acidity and increase their efficacy against *H. pylori.* We used oil in water emulsion system to prepare a novel Curcumin Clarithromycin Nano-Emulsion (Cur-CLR-NE). The nano-emulsion was validated by dynamic light scattering (DLS) technique, zeta potential; transmission electron microscopy (mean particle size 48 nm), UV–visible scanning and Fourier transform infrared spectroscopy (FT-IR). The in vitro assay of Cur-CLR-NE against *H. pylori* was evaluated by minimum inhibitory concentration (12.5 to 6.26 µg/mL), minimum bactericidal concentration (MBC) and anti-biofilm that showed a higher inhibitory effect of Cur-CLR-NE in compere with, free curcumin and clarithromycin against *H. pylori*. The in vivo results indicated that Cur-CLR-NE showed higher *H. pylori* clearance effect than free clarithromycin or curcumin under the same administration frequency and the same dose regimen. Histological analysis clearly showed that curcumin is highly effective in repairing damaged tissue. In addition, a potent synergistic effect was obvious between clarithromycin and curcumin in nano-emulsion system. The inflammation, superficial damage, the symptoms of gastritis including erosion in the mouse gastric mucosa, necrosis of the gastric epithelium gastric glands and interstitial oedema of tunica muscularis were observed in the positive control infected mice and absent from treated mice with Cur-CLR-NE.

## Introduction

One of the important members of Epsilon proteobacteria family is *H. pylori* (Roszczenko-Jasińska et al. 2020). It is one of the ubiquitous, microaerophilic, gram −ve bacteria. The *H. pylori* infect the mucosal surface of the duodenal and stomach causing gastritis and peptic ulcer and increasing risk for stomach cancer (Zamani et al. 2018). This pathogen has a specific helical shape supporting its motility through the viscous mucus layer of the stomach (Sycuro et al. 2012). *H. pylori* has involved a critical put among infectious pathogens and it has been reported as a driving challenge due to its higher resistance to the commonly utilized drugs, higher versatility, and lower targeting specificity of the available drug (Khan et al. 2022). They could be engineered to protect drugs against stomach acidity and preferentially deliver drugs to targeted sites and enhance the cellular internalization to treat intracellular infections (Yeh et al. 2020). Oil in-water drug nanoemulsion forms drug delivery systems with high oral bioavailability (Wu et al. 2020).

Oral route is the foremost common and favored course of administration due to its effortless comfort and cost effectiveness. Oral bioavailability of drugs is emphatically impacted by their formulations (Liu et al. 2016). Nanoemulsions are considered as an perfect elective for the verbal organization of drugs since they show different preferences such as tall solubilization capacity for both hydrophilic and hydrophobic drugs due to the nearness of emulsifier based interface between oil and water capacity (Gupta et al. 2016).The gold standard for treatment of *H*. *pylori*-induced infections within the clinic is a triple therapy that’s composed of a proton pump inhibitor (PPI), an expectorant, and different antibiotics (Chey et al. 2017) The antimicrobial activity of the active ingredient of Curcumin on induced *H. pylori* bacteria in the stomach of mice, showed that anti-*Helicobacter pylori* antibodies were increased in the serum of mice following induction of disease, which was not significantly decreased with curcumin and antibiotics (Ranjbar and Mohammadi 2018). Curcumin, a major compound derived from the rhizomes of turmeric, possesses a broad pharmacological effect (Liju et al. 2011) and confers anti-*H. pylori* activity (Pattiyathanee et al. 2009).

Antimicrobial nanoemulsions are emulsified mixtures of detergent, oil, and water which have broad antimicrobial activity against bacteria, enveloped viruses, and fungi (Hwang et al. 2013). Nanoemulsion technology is an alternative candidate to overcome antibiotic resistance in pathogenic bacteria (Hassanshahian et al. 2020). The major advantages of nanoemulsions as drug delivery carriers include increased drug loading, enhanced drug solubility and bioavailability, reduced patient variability, controlled drug release, and protection from enzymatic degradation (Chime et al. 2014).

This study aimed for preparation of a nano-emulsion as a vehicle to improve the bioavailability and transport a poorly water soluble clarithromycin and curcumin. Also to protect them against stomach acidity and increase their efficacy against *H. pylori *invitro and in vivo in compere with free drugs.

## Materials and methods

### Materials

Chemicals (Clarithromycin from Abbott EPC, curcumin from Sigma-Aldrich); others chemicals and reagents utilized in the following examinations and biological experiments were received at analytical standard grade (Sigma-Aldrich), and appropriated externally additional purification.

### Methods

#### Curcumin clarithromycin nanoemulsion (Cur-CLR-NE)

##### Preparation of Cur-CLR-NE

In order to prepare Cur-CLR-NE, the method of modified ultra-sonication was employed (Laxmi et al. 2015; Mosallam et al. 2021) with some modification.

To start with, course of emulsion (30/70% O/W); Oily phase was arranged by dissolving curcumin (100 µg/mL) in coconut oil (15% v/v). Tween 80 (Surfactant 10% v/v) and Propylene glycol (Co-surfactant 10 v/v) were added to the oil phase. Aqueous phase was prepared by dissolving clarithromycin (100 µg/mL in DMSO 10% v/v and water 90% v/v) and added drop wise in oily phase utilizing a mixing homogenizer to prepare a pre-emulsion. The pre-emulsion was subjected to high shear homogenization for 30 min at 10,000 rpm and 25 °C and further subjected to high energy ultra-sonication via Bench Top Ultrasonicator, for 30 min; to finally formulate Curcumin Clarithromycin Nano-Emulsion.

##### Validation of Cur-CLR-NE

For characterization of Cur-CLR-NE, several physicochemical parameters were used including UV–visible scanning, particle size and particle size distribution at NCRRT, Cairo, Egypt. The stability of the nano-emulsion is determined by zeta potential; DLS Zetasizer Technique (PSS-NICOMP 380-ZLS, USA) was employed to detect the size distribution. Transmission Electron Microscopy (TEM) was used to measure the particle size of Cur-CLR-NE using accelerating speed (80 kV) (JEOL electron microscope JEM-100 CX), while the Fourier transform infrared spectroscopy (FT-IR) was employed to assess the function moiety **(**El-Batal et al. 2020).

##### Radiation treatments

Radiation treatments: Cur-CLR-NE samples were sealed in plastic tubes and exposure to gamma rays at dose levels of 0, 5, 10 and 20 kGy at NCRRT, Cairo, Egypt; to study the impact of gamma radiation on Cur-CLR-NE stability and their sterility.

##### Stability of Cur-CLR-NE

The mechanical investigation was carried out using centrifugation. Nano-emulsions solutions were centrifuged at 7000 rpm for 15 min; this treatment is equivalent to the gravitational effect for 1months (Iradhati and Jufri 2017). Organoleptic observations were done on the physical condition of the preparation before and after centrifugation as follow; Visual observation: Nano-emulsions stability affects the products appearance and most of the time emulsion instability can be observed directly by the naked human eye. In this sense, visual observation is probably the simplest, cheapest, and quickest method to assess the gravitational separation of the emulsion without expensive analytical instruments (Hu et al. 2017). Storage and transport of Cur-CLR-NE during in vitro and in vivo assay has no phase separation, no precipitation and change in activity that indicates good stability. After agitation on a reciprocating shaker, there was no phase separation in nanoemulsion indicating that it has good stability and can withstand the mechanical forces during the transportation and handling (Jadhav et al. 2015).

##### Short- and long-term stability

The physical stability of this system, short- and long-term stability studies were carried out on the selected formulations. Samples were divided into three vials after production and stored at room temperature for 180 days. On days 0, 30, 60, 90, and 180, all samples were examined and centrifuged for 15 min at 7,000×*g*. Turbidity, phase separation, precipitation, drug separation, breaking, and creaming were evaluated (Sun et al. 2012).

##### Effect of different pH on Cur-CLR-NE

The prepared Cur-CLR-NE were subjected to different conditions to assess their stability against stomach and gastrointestinal digestion (Hassanzadeh et al. 2022). The pH of the nanoemulsion was adjusted utilizing either 0.2 N sodium hydroxide or 0.2 N hydrochloric acid, if essential pH (2, 4, 6, 7, 8 and 10), and finally, the nanoemulsions were put away at room temperatures (25 °C). The mean particle size, zeta-potential and PDI and of the prepared Cur-CLR-NE at different pH values was recorded.

##### Entrapment efficiency (%) determination

The concentration of unentrapped drug (free drug) in the formulation was measured to establish the percentage drug entrapped efficiency. This is important because it affects the drug molecule’s release properties. After separating the entrapped drug from the nanoemulsion formulation, the following Eq. (1) was used to calculate the amount of drug encapsulated per unit weight of formulation:

1 $$\begin{gathered} \% {\rm{Entrapment}}\,{\rm{Efficiency }} = {\rm{ }}({\rm{amount}}\,{\rm{of}}\,{\rm{drug}}\,{\rm{added}} \hfill \\ - {\rm{free }}\left( {{\rm{unentrapped}}} \right){\rm{ drug}})\,/\,\left( {{\rm{amount}}\,{\rm{of}}\,{\rm{drug}}\,{\rm{added}}} \right)\, \times \,100. \hfill \\ \end{gathered}$$

The entrapment efficiency of the formulated nanoemulsions was demonstrated using the centrifugation method. The concentration of drug of was determined by spectrophotometrically assay at λ max 380 nm for clarithromycin (Qamar 2014) and at λ max 460 nm for curcumin (da Silva-Buzanello et al. 2015).

##### Drug content determination

The CLR and Curcumin content (total concentration) in the nanoemulsion suspension was calculated after determining the drug concentration compared to the standard solution of CLR and Curcumin (µg/mL). To perform the determination of the drug content the nanoemulsion was appropriately diluted (200 times) with methanol and phosphoric acid 1% (50:50, v/v;) for the extraction of the drugs from the formulation matrix (Vaz et al. 2020). After centrifuge, sample of supernatant were measured at λ max 380 nm for clarithromycin and at λ max 460 nm for curcumin using UV–VIS spectroscopic method. Results were taken in triplicate and the average was taken in to consideration (Shaikh et al. 2019). The CLR and curcumin recovery was calculated as the percentage of the total drug concentration found in the nanoemulsion in relation to the initially added amount.

##### In vitro release

The release profile of the curcumin and clarithromycin from Cur-CLR-NE were examined in PBS (intestinal and mucosal pH 7.2 and gastric juice pH 1.5) at 37 °C as previously described (Li et al. 2015) with a few modification. In brief, 1 mL of Cur-CLR-NE was added to a preprocessed dialysis pack in 5 mL of PBS. Fifty-microliter samples were collected at 0, 0.25, 0.5, 1, 5, and 10 h. The concentration of curcumin and clarithromycin was recognized by spectrophotometrically at λ max 380 nm for clarithromycin and at λ max 460 nm for curcumin. The absorbance obtained was at that point converted to its corresponding concentration employing a calibration curve, and after that the precise amount of the drugs within the formulation was calculated (Malik et al. 2022).

#### *Microorganism*

*Helicobacter pylori* strains: In this study, 4 clinical isolates of *H. pylori* were used. The Four strains were isolated from biopsies of the gastric ulcer cases (October 6 university hospital, Giza, Egypt). The *H. pylori* (ATCC43504) were used as stander strain. The clinical isolates of *H. pylori* were cultivated on Columbia horse blood agar with DENT supplement (Oxoid, UK) and incubated micro aerobically (5% CO_2_, 10% O_2_, and 80% N, 120 h, 37 °C). Gram-stained bacteria were used to assess the colonies that formed on the agar plates for morphology (such as spiral shape) as well as oxidase and urease activity (Foegeding et al. 2016; Zamani et al. 2018). Spiral shaping, urease, and oxidase-positive colonies were considered typical *H. pylori* strains.

#### *In vitro determination of anti-H. Pylori*

##### Antimicrobial susceptibility testing

Curcumin Clarithromycin Nano-Emulsion was prepared aseptically at 100 µg/mL. The agar well diffusion assay was used as approved assay to test the activity of antibacterial agents against *H. pylori* (Al Somal et al. 1994; Hachem et al. 1996; Lang and García 2004) with some modification. A sterile cotton-tipped swab was dipped into the *H. pylori* suspension (2.0 McFarland) and streaked in three directions across a Mueller–Hinton agar plate containing 5% sheep blood. The plates were dried for 10 min, and then a sterile corkborer was used to drill 6 mm diameter wells into the agar after inoculation. The plates were loaded with 50 µL of Curcumin Clarithromycin Nano-Emulsion, Curcumin, clarithromycin (100 µg/mL) and incubated for 5 days in a microaerobic atmosphere at 37 °C. Curcumin and clarithromycin (100 µg/mL) was used as negative and positive control respectively. At the end of the incubation period, the diameters of the zones of growth inhibition were measured to evaluate the antibacterial activity. The negative control was WFI, whereas the positive control was a clarithromycin disc (15 µg).

##### Minimum inhibitory concentration (MIC)

The MIC is characterized by the least concentration of the antimicrobial agent capable of inhibiting bacterial development. For its assurance, the microdilution strategy was carried out in a 96 well microplate, concurring to the technique prescribed by the Clinical and research facility benchmarks Founded (CLSI) (Manyi-Loh et al. 2010; Santiago et al. 2022). The four (4) isolates and one (1) reference strain of *H. pylori* recovered from patients with gastric ulcers were selected for the determination of MICs. Briefly, different concentration of Curcumin Clarithromycin Nano-Emulsion the starting concentration was 100 µg/mL while final concentration was 0.390 µg/mL was adjusted and the bacterial inoculum was adjusted to produce the bacterial suspension 1 × 108 colony-forming units [CFUs]/mL, corresponding to 0.5McFarland standards.

All test plates were hermetically sealed and incubated for 5 days at 37 °C under microaerophilic conditions (Njume et al. 2011). Upon completion of the incubation time, the development of microbes in broth was seen outwardly and measured utilizing an ELISA microplate peruser at 620 nm. The MIC was established as the lowest concentration that inhibits bacterial cell growth in an ELISA microplate reader (Gurunathan et al. 2015).

##### Anti-biofilm activities

Biofilm formations of tested *H. pylori* strains were developed in glass tubes. Brain heart infusion (BHI) broths supplemented with 2% β-cyclodextrin (BCD) and 0.016% dimethyl sulfoxide (DMSO) were used as blank and control, respectively. After 5 days of incubation, all culture medium was evacuated. The test tubes were washed twice with phosphate buffer solution (PBS) and then dried for 30 min at 60 °C. Ten mL of 0.1% crystal violet was included for 5 min. Unbound stain was disposed of and the tubes were again dried for 30 min at 60 °C. Bound crystal violet was decolorized with ethanol/acetone blend (80:20, v/v). The level of biofilm arrangement was evaluated by measuring the absorbance of the arrangement at 570 nm employing a spectrophotometer (Vetvicka et al. 2016).

#### In vivo determination of anti-*H. Pylori*

##### Institutional review board statement

Both composed educated assents and ethical approval to perform this work were essential for both collecting human tests and mice security tests. Our work was wiped Out agreement with to the rules of the World Restorative Affiliation Helsinki affirmation for ponders on human subjects. Sometime recently beginning the work, the conventions of Test collection and in vivo mice security test were changed and endorsed by Investigate Ethical Committee of Faculty of Pharmacy Port-Said College (REC.PHARM.PSU) under the Ethical approval number (REC.PHARM.PSU-2022-1).

##### Determination of the safety limit of Cur-CLR-NE

The safety limit of Cur-CLR-NE was determined by acute oral toxicity recording (LD50 value) conducted on 6- to 8-week-old CD-1 male mice weighing 30–40gm (5 mice/group). The mice received 300 µL of 200 µg/mL 100 µg/mL, 50 µg/mL, 25 µg/mL, 12.5 µg/mL, and 6.25 µg/mL, Cur-CLR-Ns orally once daily, respectively. The mortality of the mice was recorded after 48 h (Regupathy and Dhamu 1990).

##### Anti- *H*. *pylori* activity in mice

Briefly, five groups of six to 8-weeks-old CD-1 male mice (n = 10) were used in the experiment and experiments were designed to minimize animal suffering and to use the minimum number associated with valid statistical evaluation, according to the guidelines of the animal ethics committee of the institute.

The following groups were included in the study as follows: group 1, negative control, group 2, positive control group infected by *H. pylori* but received no treatment and received phosphate buffer saline instead, group 3, infected by *H. pylori* then treated by curcumin, group 4, infected by *H. pylori* then treated by Curcumin Clarithromycin Nano-Emulsion and group 5, infected by *H. pylori* then treated by clarithromycin alone. *H. pylori (clinical isolate 3)* is used in all animal experiments*.*

Animals of both control and experimental groups were being kept separately in standard conditions and were fasted for 12 h with free access to water before each inoculation. Groups (2–6) of mice were inoculated with *H. pylori* cultures harvested in PBS twice in a period of 3 days, with about 108 CFU/mouse/inoculation. Mouse groups and control groups were kept separately, with free access to water and food. Two weeks after the final inoculation, groups 3, 4, and 5 received 200 µl of 100 µg/mL of curcumin, Curcumin Clarithromycin Nano-Emulsion (1:1) and clarithromycin alone, respectively. Group 1 received an equivalent volume of PBS (phosphate buffer saline). All mouse groups were sacrificed 3 weeks post infection, animals were anesthetized by ketamine (12 mg/kg of body weight), followed by cervical dislocation for killing, and the gastric tissues will be assessed for histopathological analysis (1, 2).

##### Statistical analysis

The differences in the means of the results between untreated and treated *H. pylori* were analyzed by Student’s t test. The probability value of p ≤ 0.05 was considered significantly different.

## Results and discussions

### Validation of Cur-CLR-NE

#### Mechanism of Cur-CLR-NE synthesis

**Fig. 1:**
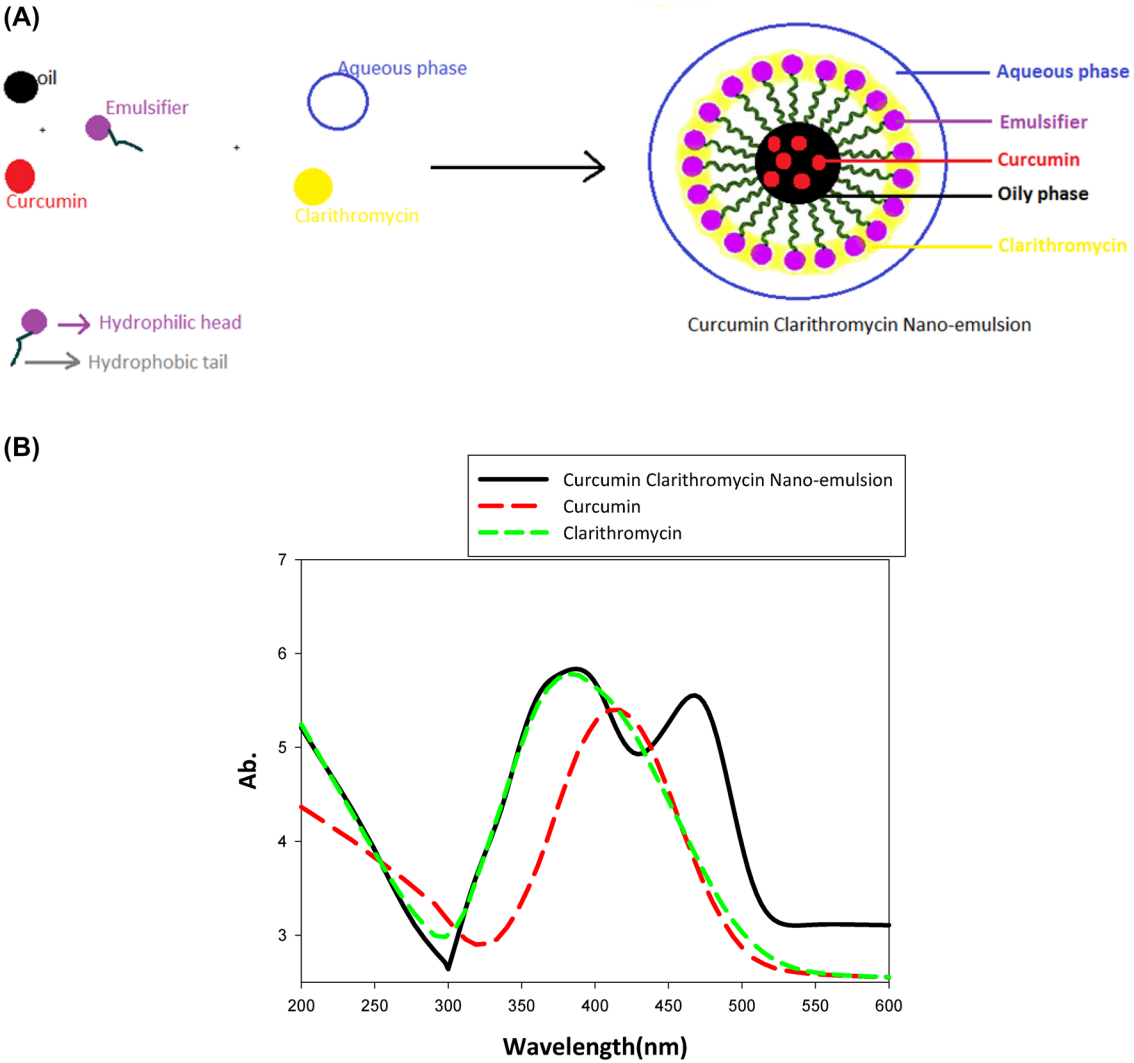
**A** Diagram of Cur-CLR-NE shape. **B** UV–visible scanning of Curcumin Clarithromycin Nano-Emulsion

The mechanism of Cur-CLR-NE synthesis by way of nanoemulsion formation is clarified in Fig. 1A under the combined activity of the curcumin and clarithromycin, the curcumin entered into the core of emulsion in oily phase and then stabilized by clarithromycin moiety that dispersed in aqueous phase by dynamic bunches of the surfactants (Fig. 1A). As a result, steady curcumin and clarithromycin of great scattering were arranged and stabilized in form of Cur-CLR-NE. Finally the curumin molecules are surrounded by two lyres of clarithromycin and surfactants molecules. Water can easily be emulsified in the solution of curcumin, clarithromycin and surfactants using homogenization and ultrasonication, this mechanism of synthesis have similarity of synthesis with previous studies (Zhao et al. 2018).

#### Optical properties of Cur-CLR-NE

The results in Fig. 1B show the optical density (OD) of Cur-CLR-NE, increases the peak intensities (OD) with two peaks in compeer with curcumin alone and curcumin Nano at λ max 380 and 460 nm that indicate presence of clarithromycin and curcumin respectively. The increased intensity and red shifts of two peaks, suggests formation of NPs with higher yields and increased sizes (El-Batal et al. 2018). In this article, we report a novel to plan Cur-CLR-NE. UV–Visible absorption spectroscopy was utilized broadly to think about this nanoemulsion. Since the band gap of the nanomaterial is greater than its bulk counterpart, the blue shift in the UV–Visible spectrum of nanomaterial is generally expected (Goswami and Sen 2012). The increase of band gap energy of nanoemulsion, through UV–Visible absorption spectra, could be a typical manifestation of size quantization in nano-solution (Calandra et al. 1999).

**Fig. 2:**
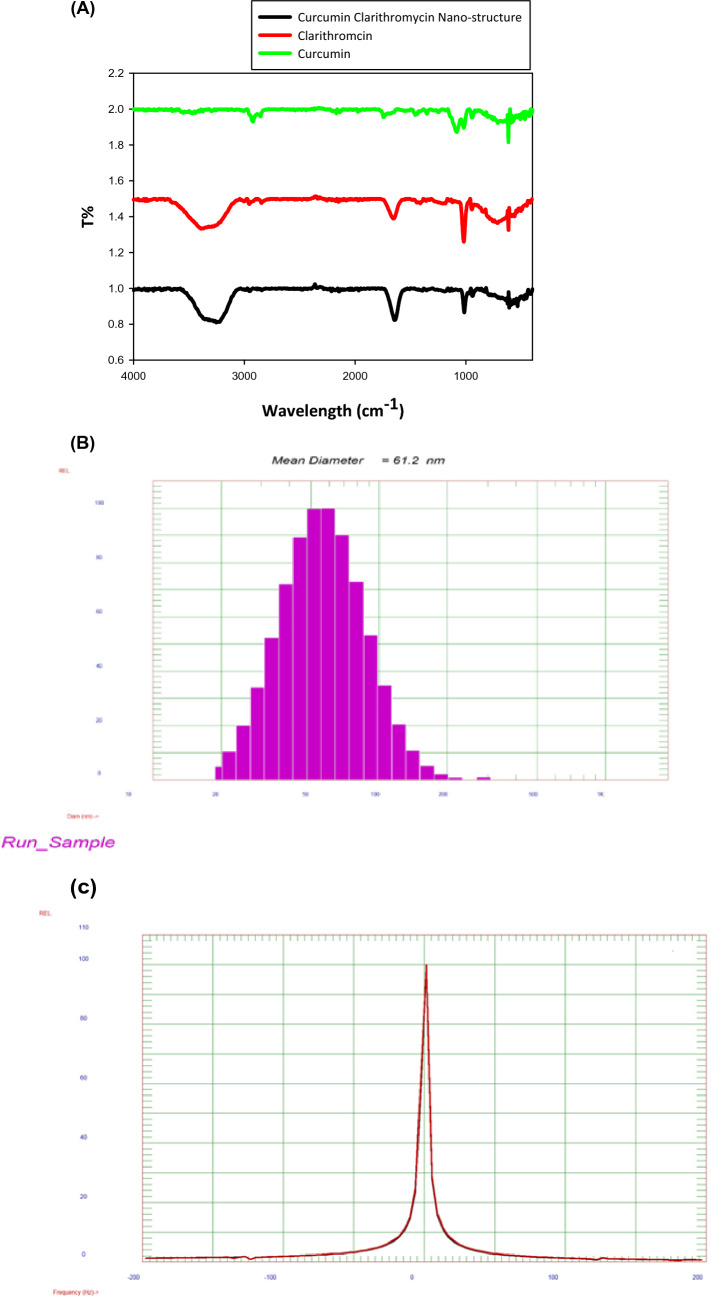

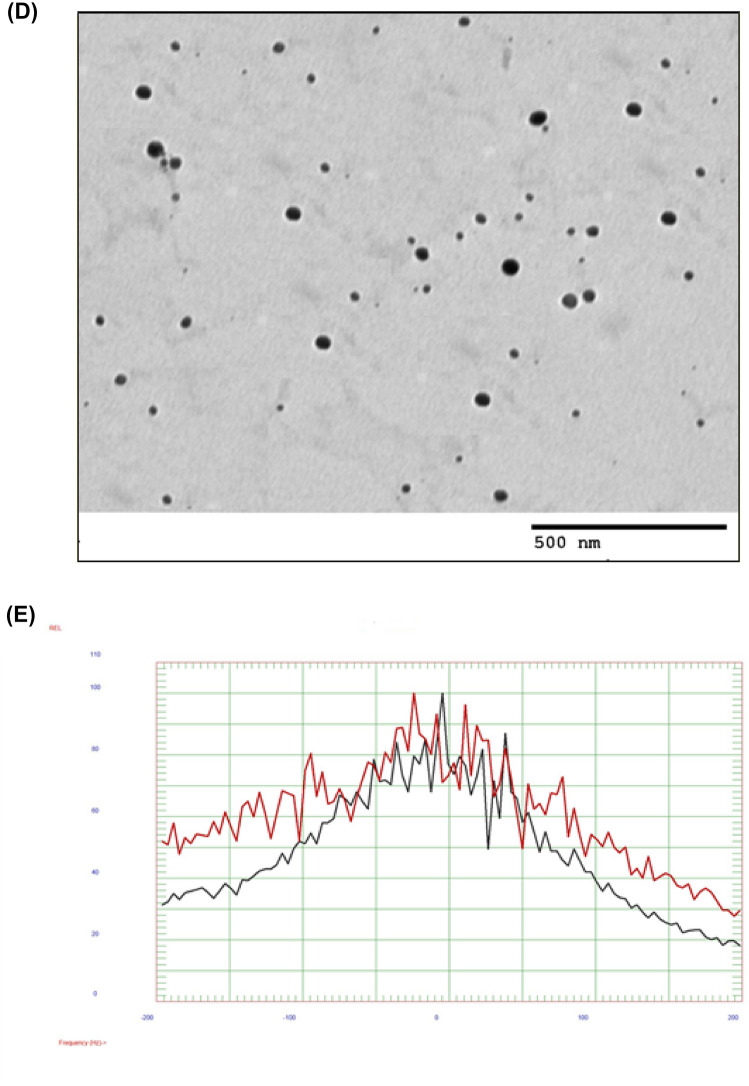
**A** Shows FTIR spectra of Curcumin Clarithromycin Nano-Emulsion clarithromycin and curcumin, **B** DLS image of Cur-CLR-NE, **C** Zeta potential of Cur-CLR-NE; **D **TEM image of Cur-CLR-NE and **E** Zeta potential of Cur-CLR-NE after gamma radiation

Figure 2A shows FT-IR spectra of Cur-CLR-NE, clarithromycin and curcumin, this analysis was conducted to determine the molecular interaction between the clarithromycin and curcumin. The spectrum in Fig. 2A shows clarithromycin; transmission at 3562 cm^−1^ assigned to the overlap of O–H vibrations; 2420 cm^−1^ to C–H bending, 2090 cm^−1^ attributed to c-o vibration and 1648 cm^−1^ to −C−O skeletal stretching. The same trend as observed in the Cur-CLR-NE spectrum (Fig. 2); for this instance, a general decrease or increase in the band with slight shifts; this attributed to stability of clarithromycin and curcumin by emulsion system. It reported earlier that could bind via the electrostatic attraction and repulsion forces, therefore, stabilization of the NEs and prevent Cur-CLR-NE separation and precipitation (Ali et al. 2021). Suggests that curcumin is introduced in the core and capped by Clarithromycin and surfactants.

#### Particle size and distribution of Cur-CLR-NE

The size distribution and zeta potential analysis of Cur-CLR-NE was performed using DLS Zeta Sizer Technique at neutral pH and the sample diluted by distilled water. The Fig. 2B shows size distribution 61.2 nm. Be that as it may, DLS measures the hydrodynamic diameter of nanoparticles; the results affirmed DLS estimate ranges of Cur-CLR-NE to be within nano scale, where the amphiphilic NPs were encompassed by water molecules, the estimate distribution of Cur-CLR-NE is still in nanoscale. A DLS estimate range of Cur-CLR-NE was watched to be bigger than TEM measure, typically attributed to DLS, which measures the hydrodynamic distance across of particles (El-Batal et al. 2020; Manaia et al. 2017; Saha et al. 2020).

Figure 2C shows the zeta potential at range from −50 mV to 50 mV, the low polydispersity values observed for all the formulations, zeta potential determination of the prepared formulations were in range of + 0.57 ± 4.05 mV. Gohel et al. 2014 refers to the high stable nanoemulsions consisted of nonionic components, which show relatively neutral charge and the NE droplet size was 11.25 nm and the zeta potential was 0.223 mV (Gohel et al. 2014). The zeta potential is an imperative factor for assessing the steadiness of a nano-form; It may be a work of the molecule surface charge, which balances the greatness of the electro-static repugnance between particles (Mukhopadhyay 2022).

Figure 2D shows the TEM image of Cur-CLR-NE that confirms the circle shape of particles with average size about 48 nm. The presence of solubilized surfactants serving as capping and stabilizing agents controls and prevents the aggregation and agglomeration of generated NE. TEM reveals the structure of molecule from interior and gives thought approximately molecule diameter and framework structure (Salvi and Pawar 2019). The spherical isotropic shape of Cur-CLR-NE confirm the one structure shape of particles where image of TEM from the particles in a nanoemulsion revealed a homogeneous structure inside the particles (Landfester 2009). Figure 2E shows that gamma radiation has negative effect on the final product stability, radical produced from gamma rays cause destroy, phase separation and finally precipitation of clarithromycin. High and or low levels of radical generation can cause increase random movements of particles and precipitation of nanoparticles (Ali et al. 2021; El-Batal et al. 2020).

#### Stability of Cur-CLR-NE

**Fig. 3:**
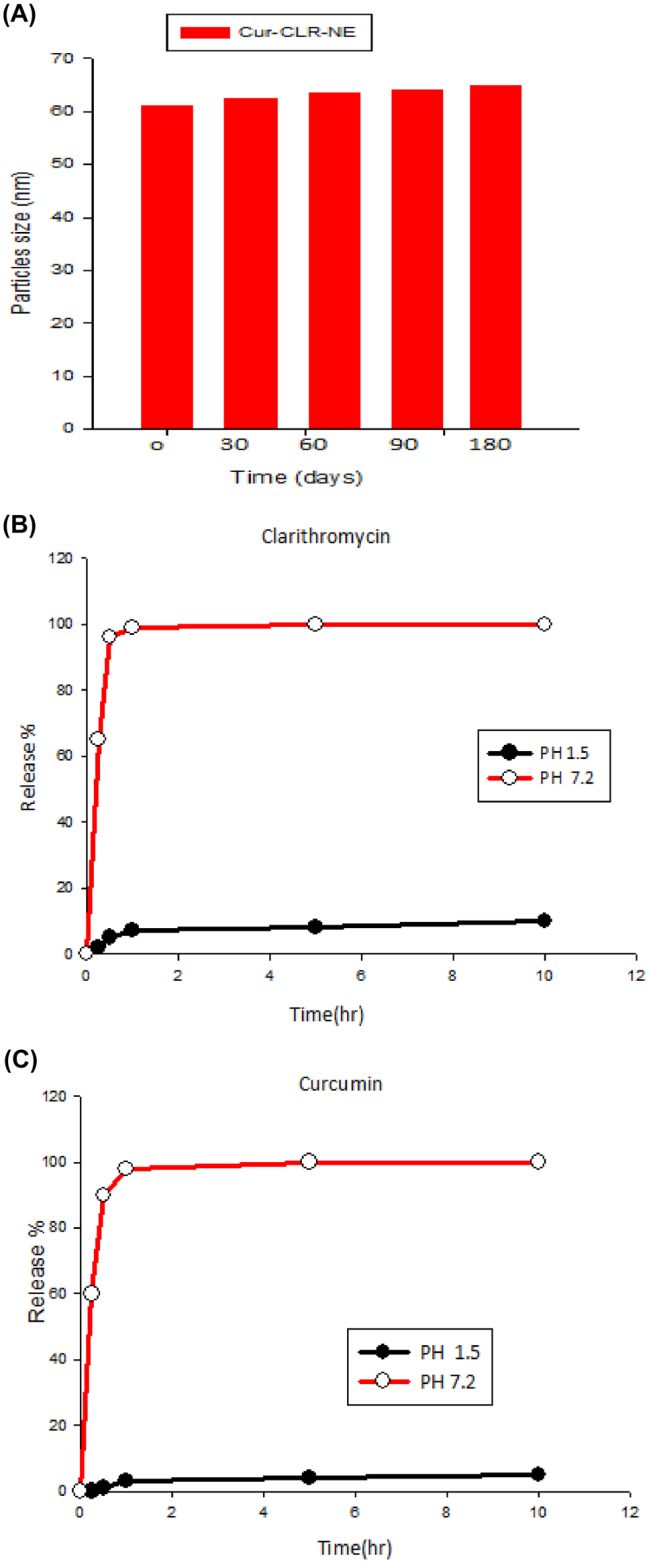
**A** Stability of Cur-CLR-NE at different time and (**b** and **c**) In vitro release of claritheomycin and curcumin from Cur-CLR-NE respectively. Recorded at 0, 0.25, 0.5, 1, 5, and 10 h. The data are expressed as the mean ± SD (n = 2). *P < 0.05, ***P < 0.001

After centrifugation on a reciprocating centrifuge, there was no phase separation in ***Cur-CLR-NE***; indicating that it has good stability and can withstand the mechanical forces during the transportation and handling. O/W NE is considered stable if the formulation maintains its physiochemical characteristics over time and under different conditions of centrifugation (Wu et al. 2020). The stable formulations of Nanoemulsions did not show any phase separation or turbidity after subjected to centrifugation (Ali et al. 2014). The main physical characteristics results particle size is shown in Fig. 3A The Cur-CLR-NE, which was stored at room temperature for 0, 30, 60, 90, and 180 days, was very stable, and the particle size of this system was within the range of 61 to 65 nm (Fig. 3A). Fluctuations in particle size were very small, and there was no change observed even after storage at room temperature for 180 days.

#### Effect of PH on Cur-CLR-NE stability

The particles have an affinity to aggregate due to appealing interfacial forces, but aggregation is at the same time stood up to by repulsive like charge interactions (McClements 2004). The mean particle size zeta-potential and PDI of the arranged Cur-CLR-NE at different pH values are appeared in Table 1. The Cur-CLR-NE had smallest particle size at acidic and neutral pH than at alkaline pH with no significant impact within the stability of zeta potential value. Since the particles carry zero net surface charge when the pH equals to the isoelectric point of the adsorbed stabilizer, which was generally the isoelectric point of curcumin (Priyadarsini 2014). The net charge of the adsorbed interfacial layer, and thus, the size of particle repulsion, is in this way highly dependent on pH (Dickinson 2010).

The droplet size and the zeta potential of the curcumin emulsions are more steady at neutral pH and acidic pH (Wu et al. 2020) where smaller emulsion droplets were produced due to contribution of smaller sized particles with a better negative charge to a better emulsion stability (Kan et al. 2023). The previous study illustrated that the alter within the initial pH of clarithromycin emulsion ranged from acidic to alkaline pH did not significantly influence drug stability (Erah et al. 1997; Lovell et al. 1995). The particle size increasing was observed at alkaline pH, our work was reliable with a past work, which recommended the emulsion droplet size increased and the zeta potential increased with the increase of pH. The reliance of the interaction potential between two nano-drugs on the interparticle separation can be modeled by summing the van der Waals attraction and electrostatic repulsion potentials (Chu et al. 2008).

**Table 1:** Mean particle size, zeta-potential and PDI of Cur-CLR-NE at different pH value

PH	DLS	Zeta potential	PDI
2	63.87 ± 1.05^b^	−8.14 ± 2.02^a^	0.323 ± 0.11^a^
4	63.23 ± 2.55^c^	−9.24 ± 1.52^b^	0.403 ± 0.20^c^
6	62.34 ± 3.55^c^	−10.64 ± 1.72^b^	0.431 ± 0.31^f^
7	61.2 ± 2.15^a^	+ 0.57 ± 4.05^c^	0.245 ± 0.01^b^
8	70.41 ± 1.43^f^	+ 26.31 ± 3.05^d^	0.720 ± 0.21^e^
10	100.62 ± 4.05d^e^	+ 30.65 ± 1.95^f^	0.922 ± 0.06^g^

Value are means ± standard deviations (n = 5)

^a–g^Different letters show statistically significant differences between conditions, where the comparison tests were performed for values of similar responses (p < 0.04)

#### Percent entrapment efficiency

The entrapment efficacy % Cur-CLR-NE of clarithromycin was 99 ± 1% on average and curcumin was 98 ± 2%. This showed good drug-loading capability for the prepared Cur-CLR-NE, which was an important requirement for the nanoemulsion (Khan et al. 2021). The higher drug entrapment was due to formulation of clarithromycin and curcumin in nanoemulsion system. The major focal points of nanoemulsions as drug delivery carriers incorporate expanded drug loading, enhanced drug solubility and bioavailability, reduced patient changeability, controlled drug release, and protection from enzymatic degradation (Chime et al. 2014).

#### Drug content determination

The formulations containing clarithromycin and curcumin showed an amount of 100 ± 0.9 µg/mL. The higher drug content of the *nanoemulsion* was due to incorporation of clarithromycin with curcumin in the double emulsion system. This showed good drug content capability for the prepared Cur-CLR-NE, which was an important requirement for the nanoemulsion (Khan et al. 2021).

#### In vitro release of Cur-CLR-NE

The gastric juice (pH 1.5), gastric mucosa and small intestine (pH 7.2) was analyzed for % release content of Clarithromycin and curcumin from Cur-CLR-NE (Fig. 3b and c). The results indicated that the Clarithromycin and curcumin was very stable and not released from the nanoemulsion by the action of acidic media. During gastric pH (1.5), over 90% of the Clarithromycin and curcumin was retained in nanoemulsion (Fig. 3b). The resistance of nanoemulsion toward gastric pH may be attributed to the stability of system in acidic pH. Stability of emulsion droplets in the gastric environment is a desirable attribute for an emulsion system in order to protect the entrapped drugs from the harsh gastric environment (Anal and Singh 2007).

But in case of pH (7.2), resulted in destabilization of the nanoemulsion and approximately 98 and 95% of the clarithromycin and curcumin respectively, was released within 15 min of incubation (Fig. 3c). Alkaline media may change the interface which facilitates release of clarithromycin and curcumin from nanoemulsion system or alkaline pH cause, coalescence between emulsion droplets seemed to occur, resulting in oiling off and breakdown of the emulsion system.

The cumulative release of claritheomycin is faster than curcumin from nanoemulsion, this due to dispersion of clarithromycin in outer aqueous phase and curcumin in oily core of nanoemulsion. Among these delivery systems, emulsions have demonstrated to be an effective and safe strategy (Yang et al. 2019). Presence of Clarithromycin in nanoemulsion form improve it stability in acidic pH and has an incredible potential for clinical applications and can be created on an industrial scale (Lu et al. 2009). Teixe-Roig et al. 2022 refer to Nanoemulsion system is significantly preventing curcumin degradation during gastric digestion (Teixé-Roig et al. 2022). For instance, utilize of Nanoemulsion based delivery system loaded with antimicrobials agent, which shows low solubility in water and is easily destroyed in acidic situations, for the eradication of *H. pylori* (Lai et al. 2022). 

### In Vitro activity of Cur-CLR-NE against *H. Pylori*

#### *H. pylori* susceptibility

All samples of Cur-CLR-NE, Curcumin, and clarithromycin were most active at 100 µg/mL against four *H. pylori* isolates and stander strain at the same concentration. The positive antibiotic clarithromycin and negative WFI controls were examined by the same procedure. The diameters of each extract’s zones of inhibition were measured, averaged, and recorded in millimeters as the mean value. The diameter of the clear zones around the wells was measured, and the results are appeared in Table 2. It can be seen that Cur-CLR-NE has a great antibacterial activity than alone curcumin and clarithromycin against all four *H. pylori* isolates and stander strain with maximum zone diameter ranged from 23 to 29 mm. Negative control exhibited no activity. The other studies confines the antibacterial activity is lowered by diffusion with longer time (Somal et al. 1994).

**Table 2:** Inhibition zone diameter (mm) of Cur-CLR-NE, Curcumin, and clarithromycin against *H. pylori* isolate and stander strains

Strains	Tested samples (100 µg/mL) zone diam. (mm)		
	Curcumin	Clarithromycin	Cur-CLR-NE
*H. pylori* ATCC 43,504 (stander)	10	13	29
*H. pylori* (clinical isolate 1)	13	14	25
*H. pylori* (clinical isolate 2)	11	16	26
*H. pylori* (clinical isolate 3)	9	16	28
*H. pylori* (clinical isolate 4)	12	10	23

#### Minimum inhibitory concentration (MIC)

The assurance of the MIC of Cur-CLR-NE comes about of 12.5, 6.25, 25, 6.25 and 12.5 µg/mL against *H. pylori* ATCC 43,504 (stander strain) and *H. pylori* (clinical isolate), separately. Cur-CLR-NE was capable to inhibit the growth of *H. pylori* at an MIC value of 6.25 to 12.5 µg/mL as shown in Table 3. The results confirm that Cur-CLR-NE can inhibit growth of *H. pylori* at concentration lower than that occur with curcumin and clarithromycin. In their study, (Baltas et al. 2016) considered natural products has great anti-*H. pylori* when MIC value is lower than 100.0 µg/mL, good is up to 500.0 µg/mL and weak activity when the concentration up to 1000 µg/mL. The study of Abouwarda et al. (2022) (Abouwarda et al. 2022) discussed the MICs of clarithromycin against *H. pylori* is at range 12.8 µg/mL and the study of De et al. (2009) (De et al. 2009) talked about the MIC of curcumin against *H. pylori is at* ranged of 50 µg/mL. The results obtained within the present study, Cur-CLR-NE appeared promising anti-*H. pylori* action, against *H. pylori* (ATCC 43,526) and *H. pylori* (clinical isolate), the activity was great. Numerous studies have ascribed a wide range of activities to curcumin and may give an appropriate premise for new used for *H. pylori* treatments (Mahady et al. 2002). To our knowledge, this study is the first to formulate curcumin and clarithromycin in form of Cur-CLR-NE and test its in vitro antimicrobial activity of Cur-CLR-NE against *H. pylori*.

**Table 3:** Minimum inhibitory concentration of Curcumin Clarithromycin Nano-Emulsion against *H. pylori*

Strains	Tested samples MIC (µg/mL)		
	Curcumin	Clarithromycin	Cur-CLR-NE
*H. pylori* ATCC 43,504 (stander)	100	50	12.5
*H. pylori* (clinical isolate 1)	50	25	6.26
*H. pylori* (clinical isolate 2)	100	50	25
*H. pylori* (clinical isolate 3)	25	25	6.25
*H. pylori* (clinical isolate 4)	100	25	12.5

#### Anti-*H. pylori* biofilm

Curcumin Clarithromycin Nano-Emulsion was capable to inhibit the growth of *H. pylori* (clinical isolate 3 more sensitive isolate to Cur-CLR-NE) at an MIC value 6.25 µg/mL. The concentrations of Cur-CLR-NE at sub-inhibitory level (1/2, 1/4, 1/8, 1/16, 1/32 and 1/64 MIC) were used for biofilm assay for *H. pylori* (clinical isolate 3). Their inhibitory effects on biofilm formation of *H. pylori* are shown in Table 4. In the control, both pellicle and attached biofilm was firstly detected in *H. pylori* since 72 h, following by an expansion of bacterial biofilm on the continuous days. The fully mature biofilm was clearly observed on 120 h and steadily maintained up to 168 h of observation. The same characteristic of biofilm production appeared in Cur-CLR-NE treated at 1/8, 1/16, 1/32 and 1/64 MIC. While, Cur-CLR-NE at 1/4 MIC markedly inhibited biofilm formation. The complete inhibition of biofilm formation was demonstrated at 1/2 MIC.

**Fig. 4:**
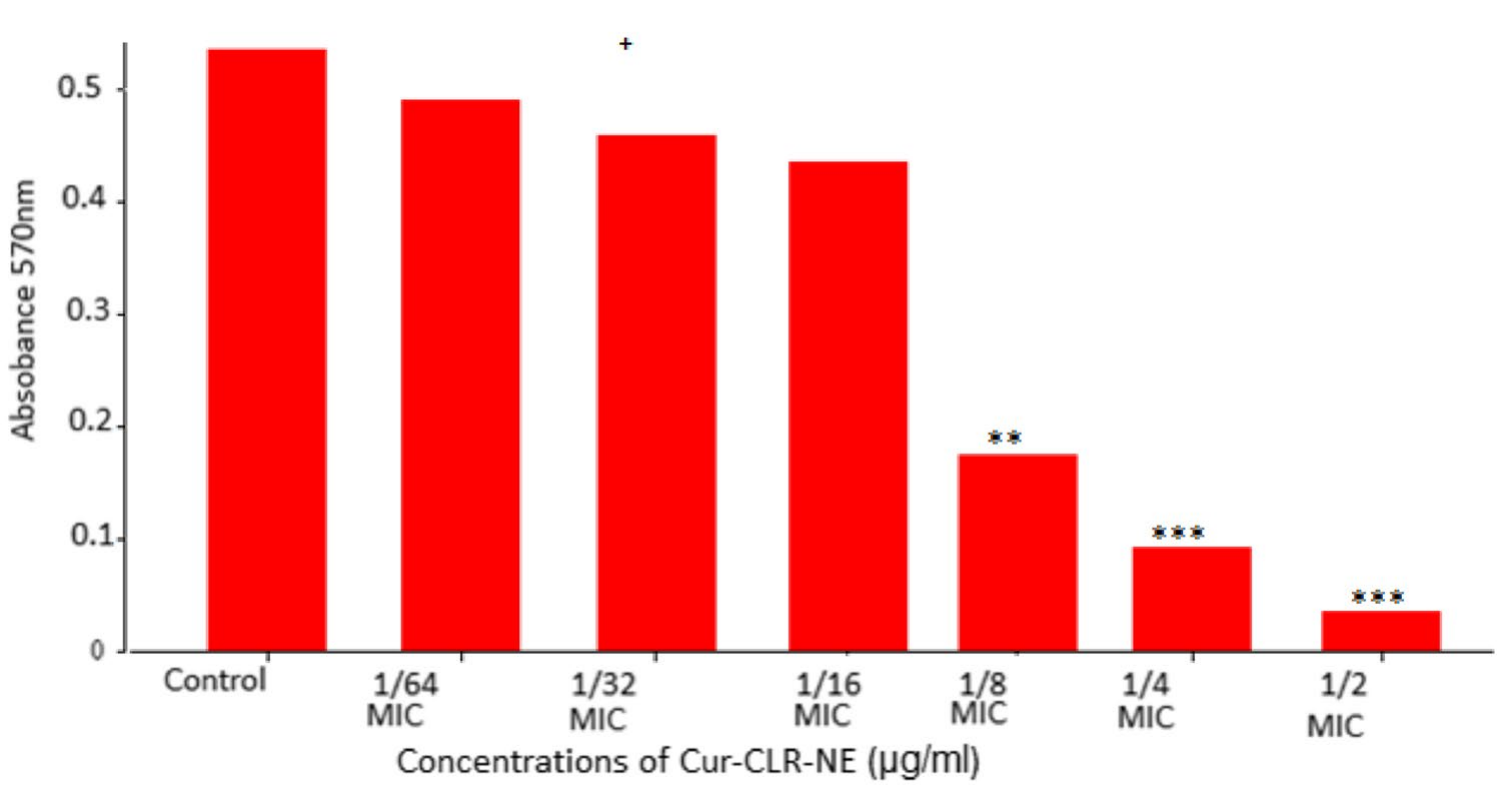
Quantified level of *H. pylori *biofilm treated with Cur-CLR-NE at subinhibitory concentrations (1/2-1/64 MIC). The data are expressed as the mean ± SD by Student’s t-test. *P < 0.05; **P < 0.01; ***P < 0.001. ‘ns’ represents no significant difference (P > 0.05)

We quantified level of *H. pylori* biofilm treated with Cur-CLR-NE at sub-inhibitory concentrations (1/2 − 1/64 MIC). As shown in Fig. 4, Cur-CLR-NE significantly decreased the biofilm formation in concentration dependent manner. The level of biofilm was markedly decreased when treated with Cur-CLR-NE at 1/2 MIC, 1/4 MIC and 1/8 MIC. In contrast, the biofilm levels were not significantly different between Cur-CLR-NE treated at 1/16, 1/32 and 1/64 MIC in comparison with the untreated one.

Generation of biofilm by *H. pylori* may be vital for enabling its resistance to antimicrobials and have defense factors(Beek and Craen 1999). Medications with standard antibiotics are incapable at eradicating biofilm related infections. In addition, the advancement of bacterial resistance is very quick which confines utilize of newer generations of antimicrobials agents from natural source as curcumin (Pattiyathanee et al. 2009). Hence, the new strategies to overcome biofilm diseases have been proposed. In later years, nanomaterials have too been utilized to kill *H. pylori* biofilms and minimize drug resistance (Arif et al. 2021) Nanodrugs made of berberine derivatives and rhamnolipids penetrated the mucus layer and effectively cleared *H. pylori* biofilms in vitro and in vivo (Shen et al. 2020). It is noteworthy that a few of the natural products tested for anti-biofilm and antibacterial ability were carried out using *H. pylori* strains that were resistant to one or more drugs (Jia et al. 2022). Grande, et al., (2020) confirm the combination of Silver Ultra-NanoClusters appeared potential synergism with metronidazole and clarithromycin with the biofilm eradication was gotten after treatment with 2x, 3x, and 4x MIC values.

A combination of curcumin and blue light light for more than 6 min disturbed *H. pylori* develop biofilms by more than 50% and upgraded the antimicrobial impact (Darmani et al. 2020). Prove from earlier studies has shown that *H. pylori* annihilation treatment requires a combination of diverse anti-microbials such as clarithromycin, levofloxacin, amoxicillin, metronidazole, and tetracycline (Graham 2009). Myricetin was the as it were natural product that synergized with all five traditional anti-*H. pylori* anti-microbials to disturb the transition of *H. pylori* from spiral to coccoid shapes (Krzyżek et al. 2021). Natural products have incredible potential to combat *H. pylori* biofilms and to address the problem of drug resistance in *H. pylori* (Hou et al. 2022).

**Table 4:** Effects of concentration of Curcumin Clarithromycin Nanoemulsion at sub-inhibitory concentrations (1/2 − 1/64 MIC) on *H. pylori* biofilm formation. Development of attached biofilm was every 24 h observed for 168 h

Time (h)	Concentration of Curcumin Clarithromycin Nano-Emulsion (Attached biofilm)						
	0	1/64	1/32	1/16	1/8	1/4	1/2
24	−	−	−	−	−	−	−
48	−	−	−	−	−	−	−
72	+	+	+	+	−	−	−
96	++	++	++	++	+	−	−
120	+++	+++	+++	+++	++	+	−
144	+++	+++	+++	+++	++	+	−
168	+++	+++	+++	+++	++	++	−

Where; Levels of individual biofilm observations are represented as: − absent; + just visible; ++ intermediate; and +++ extensive

### In vivo activity of Cur-CLR-NE against *H. pylori*

#### Histological analysis of mouse gastric tissues during ***H. pylori*** infection

During the safety limit evaluation, no dead mice were observed throughout the experimental period, indicating that Cur-CLR-Ns are safe.

**Fig. 5:**
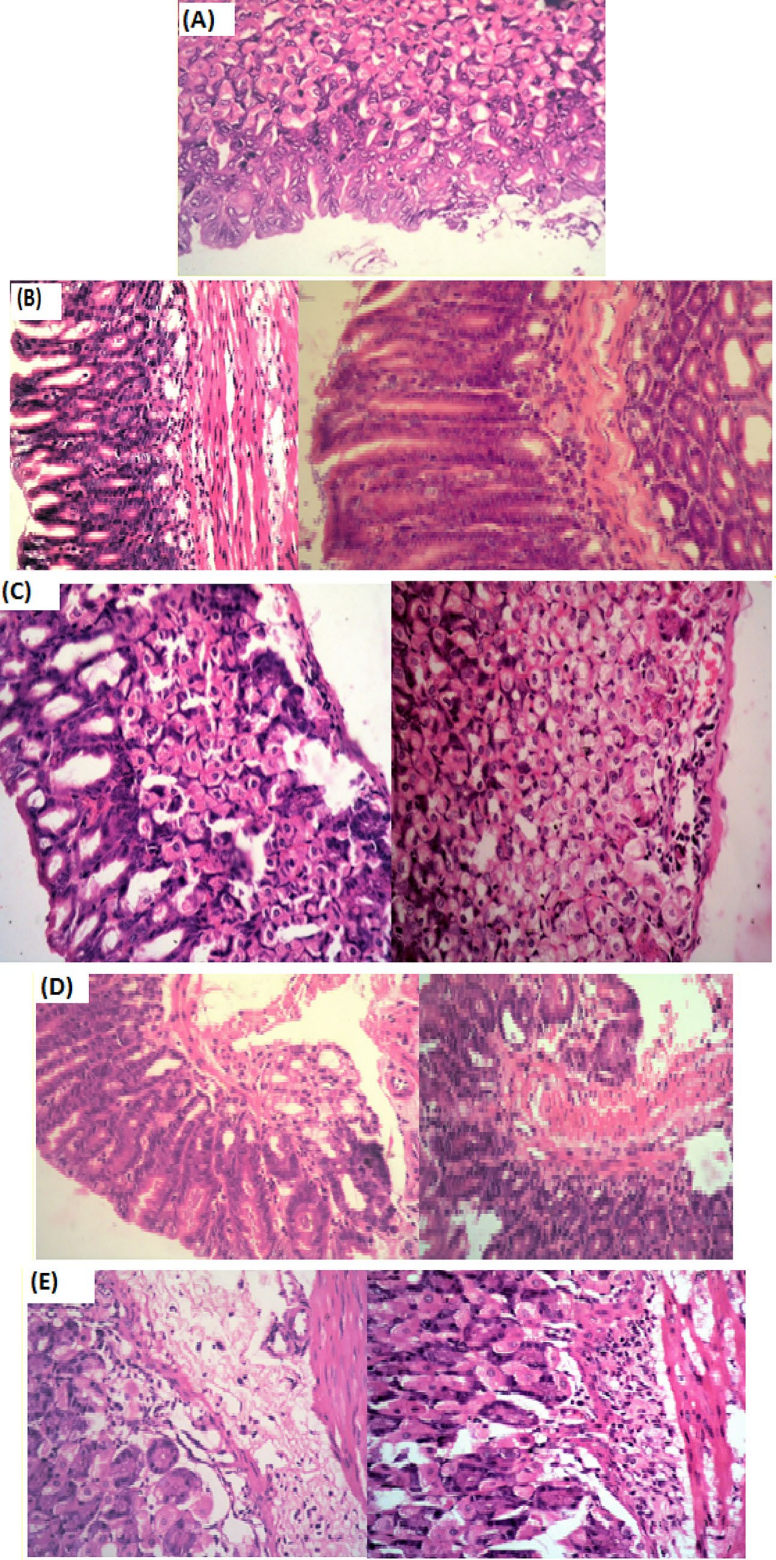
**A **Group 1: Negative control Normal stomach histological structure (H&EX 400); **B** Group 2: Positive control Sections of gastric tissues from mice challenged by *H. pylori* without any treatment, showed necrobiotic changes of gastric glands, inflammatory cells infiltmouseion and erosion of gastric epithelium of gastric mucosa, Showing infiltration of inflammatory cells in the lamina propria, necrobiotic Changes of the gastric glands. (H&E X400); **C **Group 3: Curcumin only stomach G3 little evidence of inflammatory Cell infiltration (H&E X400) Stomach (GA) showing infiltration of inflammatory deep in the gastic mucosa as well as in the gastric submucosa mainly neutrophils , **D **Group 5: Cur-CLR-NEStomach (G5) Showing vacuolations of the gastric glandular epithelium (right) with inflammatory cell mild infiltrations.  (H&E X400) stomach (G5) small areas of hemorrhages deep gastric mucosa admixed with in the few inflammatory cell infiltrations (H&E X400) and **E **Group 6: Clarithromycin Stomach (G6) showing odema and inflammatory cell infiltration deep in the gastric mucosa as well as in the submucosa. (H&E X400), Stomach Showing inflammatory cell infiltration deep in the gastric mucosa and submucosa. Moreover, necrobiotic charges of the gastric glands evident (H&E X400)

The five groups of mice were intragastrically inoculated with *H. pylori* strain while the control group received sterile phosphate buffer saline (PBS). Two weeks after the final inoculation, a groups of *H. pylori*-infected mice (3, 4 and 5) different treatments once daily for 7 days consecutively and sacrificed. Treatments were curcumin, Cur-CLR-NE and clarithromycin alone for groups 3, 4 and 5 respectively. Macroscopic examination revealed marked weight loss, lowering activity and loss of appetite. Microscopic observations revealed erosion in the mouse gastric mucosa, necrosis of the gastric epithelium and gastric glands, inflammatory cell infiltration of lamina propria and interstitial oedema of tunica muscularis of infected with *H. pylori* strain (Fig. 5B) compared to the negative control (Fig. 5A). Regarding negative control, the wall of the stomach is formed of the usual mammalian four layers as follows: Mucosa, sub-mucosa, musculosa and serosa. The mucosa cell layer is thick and folded; the thickness of the mucosa cell layer is due to the large number of glands occupying the lamina propria.

The mucous is formed of Simple columnar epithelium, Goblet cells, Lamina propria, and Muscularis mucosa (Fig. 5A). Erosion of gastric epithelium of gastric mucosa was almost abolished in all treated groups except for curcumin treated group in which degree of erosion only lowered to some extent (Fig. 5C). All treated groups showed total abolishment of gastric epithelium necrosis which was highly observed on positive control group (Fig. 5B). Only Cur-CLR-NE and clarithromycin alone could obliterate disruption and necrosis of gastric glands. Curcumin Clarithromycin Nanoemulsion was the solitary treatment strategy able to diminish inflammatory cell infiltration in the lamina propria (Fig. 5D).

These results indicated that Cur-CLR-NE showed a more complete *H. pylori* clearance effect than clarithromycin alone or curcumin alone under the same administration frequency. The results confirm the gastric mucosal damage of infected mice after treated with curcumin (Fig. 5C) and clarithromycin alone (Fig. 5E) was more severe than that treated with Cur-CLR-NE (Fig. 5C). The highest abolishment of inflammatory signs was accomplished by Cur-CLR-NE as almost all signs were totally recovered except for oedema of tunica muscularis which we recommend elongation of animal model duration to allow oedema to be totally treated. As inflammation and superficial damage usually accompany *H. pylori* infection, the symptoms of gastritis including erosion in the mouse gastric mucosa, necrosis of the gastric epithelium and gastric glands, inflammatory cell infiltration of lamina propria and interstitial oedema of tunica muscularis were observed in the infected mice. This is the first study investigating the synergistic effect between curcumin and clarithromycin in form of Cur-CLR-NE.

The role of curcumin in *H. pylori* infection was the study of interest in several research groups (Al-Rohaimi 2015; De et al. 2009; Flora et al. 2013; Nehra et al. 2022). However, comparable study between curcumin, clarithromycin and Cur-CLR-NE to investigate the synergistic effect, had to be done. For extremely hydrophobic agents like curcumin, Nanoparticle-based delivery systems will most likely be appropriate evading the drawbacks of poor aqueous solubility. Nanocurcumin has found to have more anti-inflammatory property and gives more protection to oxidative stress and apoptosis (Nehra et al. 2015). The previous information is of monstrous importance for the advancement of elective treatment against H. pylori disease since considers on high dosages of curcumin in animals and people have affirmed a need of any poisonous side effects (Goel et al. 2008).

Clarithromycin may be a key component of numerous therapeutic regimens prescribed for *H. pylori* eradication. Recently, there has been an increment in primary resistance of *H. pylori* to Clarithromycin and indeed more in secondary resistance among separates recouped from patients already treated (Branca et al. 2004). In this setting, combination therapies based upon the administration of natural compounds decreased concentrations of Clarithromycin and might have a double effect on the efficacy against antibiotic-resistant *H. pylori* strains and possibly control the improvement of resistance (Nostro et al. 2006). Finally; Histological analysis clearly showed a potent synergistic effect was obvious between clarithromycin and curcumin combination in form of Cur-CLR-NE and it’s profoundly successful in repairing harmed tissue and eradication of *H. pylori* strains.

#### Anti- *H. pylori* mechanism of Cur-CLR-NE

Antimicrobial nanoemulsions are surfactant containing oil in water emulsions which are exceptionally compelling against numerous microbes, virus, fungi, and spores at concentrations that are non-irritating to skin or mucous membranes of animals (Donovan et al. 2000). The antimicrobial action of nanoemulsions are accepted to operate as a result of the capacity of the nanoemulsions to combine with the external layers of microorganisms, with the electrostatic interaction between the cationic charge of the nanoparticles and the anionic charge on the microorganisms eventually destabilizing the membrane’s lipid bilayers and its cellular permeability, leading to disturbance (Hwang et al. 2013); subsequently the broad spectrum action of these particles.

Several drugs were assessed for the treatment of H. pylori infection, and no drug alone was successful in treating this micro-organism (Bahrami et al. 2013). Curcumin in combined therapy had synergistic effects and caused a more significant *H. pylori* reduction effect (Ranjbar and Mohammadi 2018). Nanocarriers could protect antibiotics in harsh gastric environments (Zhu et al. 2022). As for the treatment for *H. pylori*, oral drug nanocarriers own advantages in dealing with the harsh acidified environment in the stomach and the hard to reach residence site of the pathogen (Zhang et al. 2020).

This study is attempted to enhance efficiency of curcumin, clarithromycin against *H. pylori* based on nanoemulsion delivery system. The results confirm formation of Nano emulsion system contains curcumin and clarithromycin has potential activity for *H. pylori* eradication. Nanoparticles have the advantages of small size, large surface area, supported release profile, and different conduct for cargo loading, in this manner an assortment of anti-microbials can be delivered for viable destruction of *H. pylori* infection (Dening et al. 2016). In a recent study, nanoparticle could effectively eradicate *H. pylori* at the site of infection via protection of amoxicillin (AMX) from the acidic environment and sustainable drug release (Luo et al. 2018). Encapsulation in the nanoparticles facilitated the controllable and sustainable release of clarithromycin for effective eradication of *H. pylori* (Qin et al. 2021).

The nanoemulsion systems of delivery for clarithromycin and curcumin likely promote their interaction with the *H. pylori* microbial cell membranes by three main routes: (1) the increased surface area and passive transport through the outer cell membrane improves the interaction with the cytoplasmic membranes; (2) the fusion of the emulsifier droplets with the phospholipid bilayer of the cell membrane likely promotes the targeted release of the essential oils at the desired sites; and (3) the electrostatic interaction of positively charged nanoemulsions droplets with negatively charged microbial cell walls increases the concentration of drugs at the site of action (Lu et al. 2018).

Cur-CLR-NE was eradicating *H. pylori* where, role of surfactant micelle in nanoemulsion improve penetration of curcumin and clarithromycin inside the *H. pylori*. Micelles are totals of surfactants self-assembled in water solution they are utilized Nano by a hydrophilic space named crown and a hydrophobic space called center which remains in contact with hydrophobic drugs such as Curcumin (Kataoka et al. 2012).

Presence of surfactants in Nano-emulsion systems can be made through emulsification, which can control the size of the drops and increment the medicate solubility and efficacy especially antimicrobials effects (Gupta et al. 2016). Clarithromycin could be a key component of numerous therapeutic regimens prescribed for *H. pylori* eradication. Recently, there has been an increment in primary resistance of *H. pylori* to Clarithromycin and indeed more in secondary resistance among separates recouped from patients already treated (Toracchio et al. 2005). Curcumin has been antibacterial impact against Gram-positive and Gram-negative species, counting strains dependable for human contaminations and showing antibiotic resistance as *H. pylori* (Praditya et al. 2019).

Advancement of Nano for entanglement and conveyance of antimicrobial substances represent an alternative to the coordinate application of these substances (Brandelli 2012). Nano materials have progressively been utilized as a substitute for antibiotics and additives in different products to confer anti-microbial impact (Baranwal et al. 2018). The complexion of curcumin with other therapeutic antibiotics in form of Nano plays an important role within the therapeutic properties; due to the diketone moiety within the Curcumin chemical structure empowers it to make complexes with other particles (Shakeri et al. 2019).

## Conclusion

Biocompatible oil in water nanoemulsion was fabricated successful as a vehicle to transport a poorly water soluble drug, clarithromycin and curcumin. Such nanoemulsions enable the lipophilic drug to be more effective and better when compared with the oil solution against *H. pylori*. This nanoemulsion system hence the bioavailability of clarithromycin and curcumin as evidenced from the increase in zone of inhibition in the anti- *H. pylori* activity assay. Furthermore, this system was stable for at least 180 day. Oral nanoemulsion shows efficiently and effectively deliver curcumin and clarithromycin via Cur-CLR-NE to the lesion sites in a controllable manner have shown some promising results. A novel Cur-CLR-NE based on H. pylori eradication and protection against its harmful effects on the stomach wall and mucosa. The Cur-CLR-NE showed a more complete *H. pylori* clearance effect than clarithromycin or curcumin under the same administration frequency. Previous properties of the novel Cur-CLR-NE nanoemulsion system posit its suitability for clinical applications of the poorly water-soluble curcumin and clarithromycin to enhance anti-H. pylori antibody.

## Data Availability

The data and materials that support the findings of this study are available from the corresponding author, upon reasonable request.

## Declarations

### Constant of participate and publish

All authors agree to participate and publish.
